# Supplementary material for: Autophagy Mediates Escherichia Coli-Induced Cellular Inflammatory Injury by Regulating Calcium Mobilization, Mitochondrial Dysfunction, and Endoplasmic Reticulum Stress
Source: Int J Mol Sci. 2022 Nov 16;23(22):14174. doi: 10.3390/ijms232214174 (PMC9698444; doi:10.3390/ijms232214174)
Supplement: Supplementary file 1 [file ijms-23-14174-s001.zip › ijms-1971059-supplementary.pdf]

Table S1: Primer pairs used for Real time quantitative PCR

Name	Sequence of primer (5' to 3')	Literature or gene number
IL-1β-F	CAACCGTACCTGAACCCA	NM_174093.1
IL-1β-R	ACGATGACCGACACCACC	
IL-18-F	ACTTTGGCAAACTTGAACC	NM_174091.2
IL-18-R	CCTCTAGTGAGGCTGTCCTT	
mtND1-F	CTAGTCTCGGGCTTCAACG	NC_006853.1
mtND1-R	GGGTAGGATGCTCGGATT	
mtND2-F	AGCCTACTCATCAATCGC	
mtND2-R	TGACAGGGTAGTGGTGGT	
MtND3-F	CATCGCATTCTGACTTCC	
MtND3-R	ATTTGCTGTTTGTGAGGC	
MtND4-F	TTACCCGATGAGGAAACC	
MtND4-R	ATAACGAGTGCTATGTGGC	
MtND5-F	TGATACGGACGAGCAGAT	
MtND5-R	TTGAGTCGCTTGGGTTTA	
MtND6-F	CTGTAGCCATAGCCGTTGT	
MtND6-R	GGGTTAGGGTTAATTGTGAGT	
POLG-F	AAGGCGAAGAAAGGGAAGA	XM_024982309.1
POLG-R	TGGAGGCTGAGGAGACTGG	
SSBP1-F	AGGCATGAGTCTGAAGTAGCT	NM_001037466.1
SSBP1-R	CGGTCGGAACACTGAAAT	
TWNK-F	CGAGGAACAGCCTGCTTA	NM_001098463.1
TWNK-R	TCCGATGTCCCTTCAAAA	
TOP-F	AGTCCGACACGATAACAAG	NM_001206487.2
TOP-R	CGATGAAGTACAGGGCTAC	
PGC1α-F	CATGGTACGTGCCATAAA	NM_001205551.3
PGC1α-R	AGAGCAGAACCGCTTGAT	
TFAM-F	AAGATGCTTACAGGGCAGAC	NM_001034016.2
TFAM-R	TGTTCCTCCCAAGATTTCA	
NRF1-F	GCCATTGTCCTTTGTATCTC	NM_001098002.2
NRF1-R	AACCCTTTGCTTCTGCTC	
GAPDH-F	ATGCTGGTGCTGAGTATGT	NM_001034034.2
GAPDH-R	CAATCTTGAGGGTGTTGTTAT	

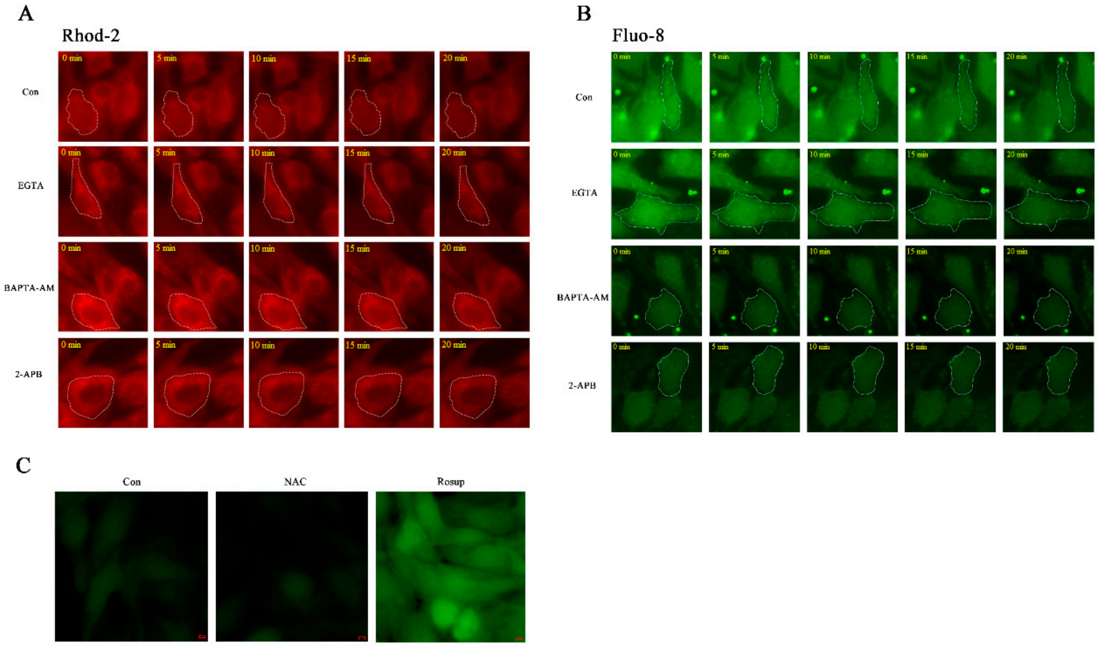

Figure S1: Effects of inhibitors on mitochondrial Ca2+, cytoplasmic Ca2+ and ROS. Primary bEECs were treated with EGTA (5 mM), BAPTA-AM (10 μM), 2-APB (50 μM) or NAC (8 mM). EGTA is a Ca^{2+} chelator that captures extracellular Ca^{2+}. BAPTA-AM is a selective, permeable free Ca^{2+} chelator that sequesters cytoplasmic Ca^{2+}. 2-APB inhibits IP3 receptors and blocks release of calcium from store-operated calcium channels. The anti-oxidant properties of NAC are thought to combat some of the effects of oxidative stress. (A) Live-cell imaging was used to monitor mitochondrial Ca^{2+} levels. (B) Fluo-8 AM was used to monitor cytosolic Ca^{2+} levels in real time. (C) ROS levels was detected by DCFH-DA fluorescence after 6 h of NAC treatment. Rosup was a positive control for ROS.
